# Effects of Fermented Soy on Cognition in Older Adults: Outcomes of a Randomized, Controlled Trial

**DOI:** 10.3390/nu17182936

**Published:** 2025-09-12

**Authors:** Laura M. West, Joan Sabaté, Ifeanyi D. Nwachukwu, Grace J. Lee, Rawiwan Sirirat, Amandeep Wright, Sujatha Rajaram

**Affiliations:** 1 School of Public Health, Loma Linda University, Loma Linda, CA 92350, USA; laurawest@llu.edu (L.M.W.); jsabate@llu.edu (J.S.); rsirirat@llu.edu (R.S.); amawright@llu.edu (A.W.); 2 Department of Public and Allied Health, Bowling Green State University, Bowling Green, OH 43403, USA; idnwach@bgsu.edu; 3 Department of Psychology, School of Behavioral Health, Loma Linda University, Loma Linda, CA 92350, USA; gracelee@llu.edu

**Keywords:** fermented soy, cognition, memory, soy, isoflavone, older adults, dementia

## Abstract

**Background/Objectives**: Soy foods and isoflavones are inversely associated with cognitive decline; however, randomized controlled trials (RCTs) show mixed results. Fermented soy contains bioactive compounds not found in unfermented soybeans, such as peptides and aglycone isoflavones, which may support cognition by reducing neuroinflammation and oxidative stress. Fermented soy RCTs on older adults with mild cognitive impairment show cognitive benefits; however, the effects of fermented soy on cognitively healthy older adults are not known. **Methods**: We investigated the effects of a non-probiotic fermented soy powder, added to the usual diet, compared to a placebo matched for energy on global cognition, memory, verbal fluency, processing speed, and executive function in a 12-week RCT on 61 adults aged 65 years and older (74 ± 5 y; 47 completers). **Results**: The fermented soy group showed significant improvement (*p* = 0.041) in memory scores (1.81%, 95% CI: −2.10, 5.72) vs. placebo (1.16%, 95% CI: −3.64, 5.97) using analysis of variance, adjusted for sex and baseline scores. Post hoc analyses on women 70 years and older (*n* = 29) found significant improvement in global cognition (*p* = 0.028) and memory (*p* = 0.049) in the fermented soy group. Global cognition mean change adjusted for baseline scores was 2.86% (95% CI: 1.52, 4.21) for fermented soy and 0.06% (95% CI: −1.43, 1.55) for placebo. Memory mean change adjusted for baseline scores was 8.47% (95% CI: 5.05, 11.89) in the fermented soy group, compared to 2.05% (95% CI: −1.75, 5.84) for placebo. **Conclusions**: These outcomes suggest that fermented soy has the potential to slow age-related cognitive decline, especially memory for women 70 and older. Further research to confirm these findings in older women, and in males and other age categories is warranted.

## 1. Introduction

Increased life expectancy has contributed to an aging population, with global dementia cases estimated to rise from 57.4 million in 2019 to 152.8 million in 2050 [1]. Nutrition and other lifestyle factors have been shown to modify the risk for cognitive decline [2]. Healthful dietary patterns containing mostly whole plant foods have shown neuroprotective effects [3,4,5,6]. These plant-focused dietary patterns are rich in antioxidant nutrients, *n*-3 polyunsaturated fatty acids, and anti-inflammatory phytonutrients including polyphenols and other bioactive compounds [7,8]. Phytonutrients are thought to support healthy cognitive and cerebrovascular function, by reducing oxidative stress and neuroinflammation, which are associated with neuronal damage and can increase the risk of age-related cognitive decline (ARCD) [7,8,9].

Widely consumed in traditional Asian diets, soy foods are rich in isoflavones (daidzein and genistein), phenolic flavonoids with antioxidant and estrogen-like properties linked to cardiovascular [10], skeletal [11], and cognitive health, especially memory [12]. Despite this potential, findings from observational studies and clinical trials on soy and cognition in older adults remain inconsistent [13,14], due to limited bioavailability of key isoflavones in their native glycoside forms. Fermentation enhances the nutritional profile of soy by converting isoflavone glycosides into aglycones, which are more readily absorbed and biologically active [15,16,17]. This process also amplifies antioxidant and anti-inflammatory properties, offering a plausible mechanism for improved neuroprotective effects.

While animal studies strongly support the cognitive benefits of fermented soy [18,19,20,21,22,23,24,25,26], human research has primarily targeted individuals with mild cognitive impairment (MCI). A 12-week trial with a probiotic fermented soybean powder improved attention and memory composites and increased brain-derived neurotrophic factor (BDNF) in older adults with MCI [27]. Similarly, a six-month tempeh intervention improved global cognition in older men and postmenopausal women with MCI [28]. To our knowledge, this is the first randomized controlled trial (RCT) to examine the cognitive effects of a non-probiotic, fermented soy product in a population of cognitively healthy older adults, addressing an important gap in the literature and expanding the potential preventive relevance of fermented soy intake.

We previously demonstrated that Q-Can Plus^®^, a fermented, non-probiotic soy product, significantly reduced LDL cholesterol [29] and lowered inflammatory markers, including interleukin-1 receptor antagonist and interleukin-6 [30], both associated with protection against brain aging [31,32,33,34]. Additionally, Q-Can Plus^®^ has been shown to modulate the gut microbiota by increasing Bifidobacteria and other beneficial bacteria [35], which are known for their neuroprotective effects [36,37] but typically decline with age [38,39]. The purpose of this study was to determine if regular intake of the fermented soy product, Q-Can Plus^®^ enhances cognitive function including global cognition, and domain specific cognitive function (memory, verbal fluency, processing speed, and executive function) among adults 65 years and older.

## 2. Materials and Methods

### 2.1. Study Protocol

**Figure 1:**
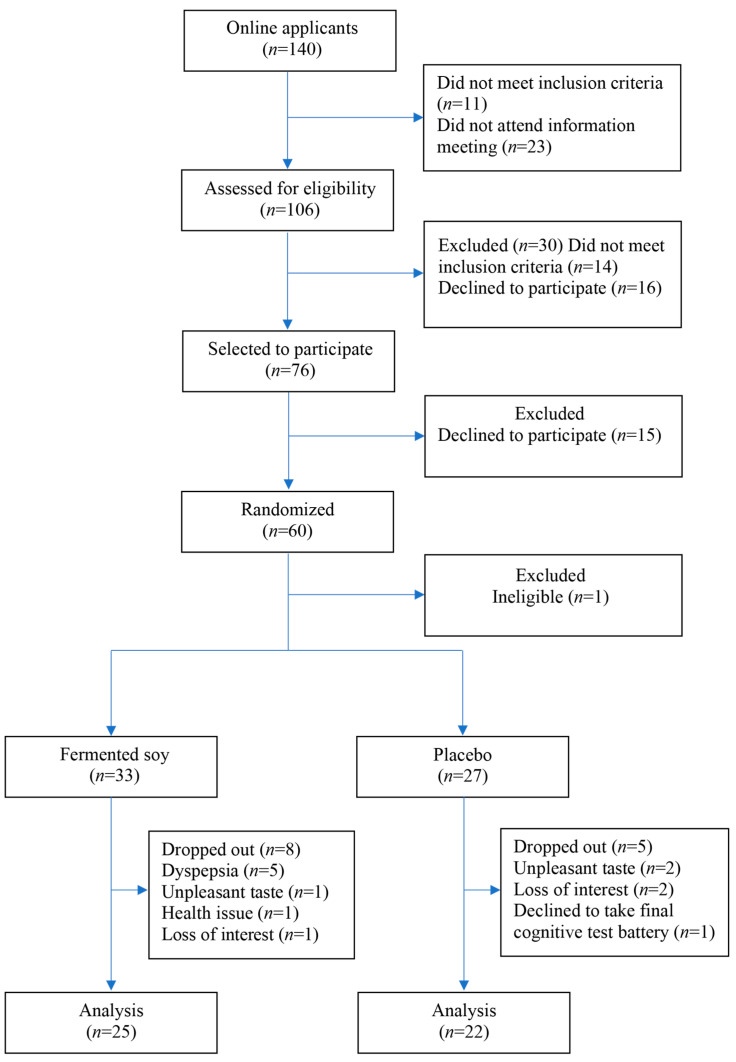
Study flowchart.

This 12-week, free-living, parallel, triple-blind, placebo-controlled RCT with healthy men and postmenopausal women 65 years and older aimed to explore the effects of a fermented soy product on immunity and cognition (ClinicalTrials.gov NCT04866576). Only the cognition outcomes will be discussed here. The Institutional Review Board of Loma Linda University approved the study, and all participants provided written informed consent prior to enrollment. Study subjects were recruited from Loma Linda, CA, USA, and the surrounding areas. Healthy men and postmenopausal women, 65 years of age and older and ambulatory were eligible for participating in the study. (Figure 1) Exclusion criteria included: (1) known intolerance or allergy to soy or dairy products; (2) immune system insufficiency/disease; (3) insulin dependent diabetes mellitus; (4) neurodegenerative disease and MCI; (5) kidney dialysis; (6) cancer radiation or chemotherapy treatment; (7) prednisone or prednisolone therapy greater than 10 mg/day within 6 months of the study. Subjects were instructed to continue their usual diet, physical activity, and other lifestyle habits, and not to take any supplement known to affect cognition and immunity for the duration of the study. We have successfully applied the recruitment and study protocols used in this study in several previous RCTs [29,40].

One hundred and forty individuals applied to participate in the study. From these, 106 were assessed for eligibility, 76 of whom were selected to participate. Sixty-three eligible individuals agreed to participate in the study and out of these, two declined to complete the cognitive test battery. The remaining 61 participants were randomized into either one of two groups: fermented soy (*n* = 33) and control (*n* = 28). Sample size was calculated for the other primary outcome, immune markers, and including a 10% dropout rate, was estimated at 62.

Selected subjects were randomly assigned to either the fermented soy or placebo group, stratified by sex. Randomization group status was blinded for participants, clinicians, and statisticians. Household members, such as partners or friends, were randomized together into the same group. After randomization, participants completed questionnaires on demographics and lifestyle habits, including diet, physical activity, stress, sleep, and alcohol, tobacco, and caffeine use. The questionnaires, except for demographics, were repeated at week 12.

Trained research clinicians measured participants’ height, weight, and body composition at baseline and week 12 visits. Body weight was measured to the nearest 100 g with an InBody^®^ 570 Body Composition Analyzer (Cerritos, CA, USA). Height was measured using a wall-mounted stadiometer to the nearest 0.1 cm.

Subjects consumed two pre-measured packets of Q-Can Plus^®^, fermented soy powder or whey-based placebo powder each day for 12 weeks. Q-Can Plus^®^, is patented by BESO Biological Research Inc. (Diamond Bar, CA, USA) and is fermented with a confidential mix of beneficial organisms, but does not contain live cultures, and thus is not a probiotic. Control subjects consumed two packets of whey-based placebo powder designed to be isocaloric and visually comparable to the fermented soy powder. The fermented soy powder contained 110 kcal, 8.4 g carbohydrate, 4.5 g fat, 9.4 g protein, 5.6 g fiber, and 36.3 mg isoflavones per day, a moderate dose of isoflavones typical of that found in traditional Asian diets [41]. In studies with MCI patients, higher doses may be warranted. The placebo powder contained 106 kcal, 15.6 g carbohydrate, 1.0 g fat, 8.5 g protein, 0 g fiber, and 0 mg isoflavones. Both fermented soy and placebo powders were flavored with cocoa and monk fruit sweetener. Participants were instructed to mix their packets with eight ounces of water or a usual beverage such as dairy milk or plant-based milk. All participants were directed to maintain their usual diet and lifestyle habits for the duration of the study and to keep consumption of soy foods to a minimum. Most participants reported on baseline surveys that they rarely or never ate soy foods and none took isoflavone supplements. Participants who reported consuming soy foods were instructed to keep consumption to a minimum for the study duration, i.e., no more than one serving per week. The participants recorded any illnesses and deviations from their intervention product intake, usual diet, lifestyle, and medications in a lifestyle journal.

Participants met monthly with the study clinician, at which time they picked up their beverage packets, returned unused packets, and completed questionnaires about their experience with and tolerance of their intervention product. Compliance with the intervention was determined by counting unused, returned packets and subjective assessment of their lifestyle journal.

A 90 min comprehensive battery of neuropsychological tests was administered to participants at baseline and week 12 by psychometrists blinded to participants’ intervention group. Participants were evaluated on the mean change in test scores from baseline to week 12 for global cognition and in the cognitive domains of memory, verbal fluency, processing speed, and executive function. To reduce variability, baseline and end-of-study cognition tests were scheduled for the same time of day and participants had the same psychometrist for both testing sessions. Participants were instructed to keep other conditions known to affect cognition, such as hours of sleep and caffeine intake, similar for baseline and end-of-study testing.

We have previously used this cognitive test battery successfully with the older adult population [42]. Cognitive tests in the memory composite included immediate and delayed recall scores for the Rey Auditory Verbal Learning Test and Brief Visuospatial Memory Test—Revised. The FAS Phonemic Verbal Fluency Test and Animals Naming Test composed the verbal fluency composite. Tests of processing speed included the Symbol Digit Modalities Test, Trail Making Test (TMT) Part A, and Stroop Test (Golden version) Parts A and B. Executive function tests included TMT Part B, Stroop Test Part C, Digit Span from the Wechsler Adult Intelligence Scale—Fourth Edition, and Auditory Consonant Trigrams. Global cognition was assessed by creating a composite of all four cognitive domains. The Center for Epidemiologic Studies Depression Scale was included to assess depressive symptoms [43].

### 2.2. Statistical Analyses

The primary outcome was to assess mean changes in global cognition and the cognitive domains of memory, verbal fluency, processing speed, and executive function, as measured by a validated battery of cognitive tests. Sample size was calculated for the other primary outcome, immune markers, and including a 10% dropout rate, was estimated at 62. Power for cognitive composite outcomes was calculated post hoc. All analyses were intention-to-treat and included all participants who completed the end-of-study cognitive test battery, regardless of level of compliance. The proportion of maximum scaling method was used to transform raw test scores to a metric from 0 (minimum possible) to 1 (maximum possible) [44]. For ease of interpretation, scores were multiplied by 100 to yield the percentage of the maximum possible score. The highest observed participant score was used in place of maximum possible for tests that did not have a maximum possible score (Stroop and TMT). TMT scores, which reflect the completion time in seconds, were reversed for inclusion in composites so that higher scores reflected faster, i.e., better, performance. Composite scores were calculated as an average of the relevant standardized scores. Two-sided tests of significance were used and a *p*-value of less than 0.05 was defined as statistically significant. Mean change in scores from baseline to post-intervention were analyzed using analysis of covariance (ANCOVA), adjusted for baseline scores and sex. All data were analyzed using SPSS version 29.0.1.0.

## 3. Results

Of the 61 participants randomized, most (*n* = 29) were postmenopausal women 70 years and older. One participant randomized into the placebo group had recently been diagnosed with MCI and hence was no longer eligible to participate. Eight participants in the fermented soy and five in the placebo group dropped out before the final cognitive testing. In the fermented soy group, five dropped out due to dyspepsia and one for unpleasant taste of intervention product, health issues unrelated to the intervention, and loss of interest in the study. In the control group, two participants dropped out for unpleasant taste of the placebo product, two for loss of interest in the study, and one for declining to take the final cognitive test. The overall dropout rate was 22%, which is consistent with other nutrition intervention trials in older adults [45,46]. Reasons for attrition were mostly related to palatability of the intervention. Forty-seven participants completed the study: 25 in the fermented soy group and 22 in the control group. The mean age was 74.0 years for completers and 71.7 years for dropouts, a non-significant difference (p = 0.148). Six out of the 13 participants who dropped out were < 70 years old. Significantly more Hispanics dropped out compared to Whites and other racial groups (*p* = 0.013). Those who completed the study and those who dropped out were similar in all other respects (Supplemental Table S1).

There were no significant baseline differences between fermented soy and control participants among completers (Table 1). Although body mass index (BMI) was not a specific criterion for participation, mean BMI was in the overweight range. Participants did not use isoflavone supplements and the reported dietary intake of soy foods was low. Baseline isoflavone intake was similar between fermented soy and placebo groups. Based on packets consumed and returned, compliance to the fermented soy and placebo interventions was estimated to be 90% (151 out of 168 packets consumed) for the fermented soy group and 93% (156 out of 168 packets consumed) for the placebo group.

### Cognition Outcomes

There were no significant differences between fermented soy and placebo groups in baseline composite scores for global cognition or specific domains of memory, verbal fluency, processing speed, and executive function (Table 2) or in baseline raw test scores (Supplemental Table S2). After adjusting for sex and baseline scores, the fermented soy group showed modest, but significant improvement (*p* = 0.041, observed post hoc power = 0.71, Cohen’s d = 1.02) in memory scores, 1.81% (95% CI: −2.10, 5.72) compared to placebo, 1.16% (95% CI: −3.64, 5.97) (Table 2). No significant between-group differences in cognitive change scores were observed in other domains, or for global cognition. Table 3 shows baseline raw scores for individual cognitive tests.

**Figure 2:**
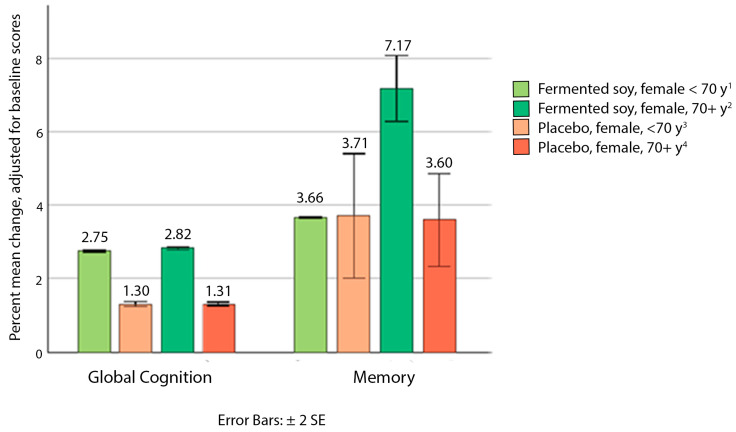
Change in global cognition and memory in postmenopausal women, younger than 70 y vs. 70 and older. ^1^ *n* = 2. ^2^ *n* = 16. ^3^ *n* = 5. ^4^ *n* = 13.

Our study group consisted of 75% women, with mostly 70 years and older, so we conducted post hoc analyses to determine if older postmenopausal women had a significantly different response to the fermented soy intervention. We noted significant differences between the fermented soy and placebo groups in women ≥ 70 years for mean change in global cognition and memory scores (Figure 2). After adjusting for baseline scores, the mean change in global cognition scores for women 70 years and older was 2.86% (95% CI: 1.52, 4.21) in the fermented soy group, compared to 0.06% (95% CI: −1.43, 1.55) for placebo (*p* = 0.028, observed post hoc power = 0.68, Cohen’s d = 1.13). Adjusted mean change in memory composite scores was 8.47% (95% CI: 5.05, 11.89) in the fermented soy group, compared to 2.05% (95% CI: −1.75, 5.84) for placebo (*p* = 0.049, observed post hoc power = 0.59, Cohen’s d = 1.02). Complete cognitive composite data for women 70 years and older can be found in Supplemental Table S3.

## 4. Discussion

In this 12-week randomized, controlled dietary intervention study investigating the effects of daily consumption of a fermented soy product on cognition in older adults, a modest, but significant improvement was observed in the memory domain. In exploratory analyses, a more pronounced effect was noted among postmenopausal women 70 years and older (8.47% mean improvement in memory scores for fermented soy group compared to 2.05% for placebo group). For comparison, a study in Greece examined normative data on verbal memory scores in older adults and found mean scores were 6.1% to 9.6% lower in the 70–79 year age group compared to the 60–69 year age group [47]. Our results are exploratory and should be interpreted with caution until confirmatory trials can be conducted.

Memory has been shown to have a stronger response to soy interventions, compared with other cognitive domains [48]. In a meta-analysis of 16 RCTs on soy isoflavone intervention and cognition, Cui et al. found memory to be the only domain showing significant improvement [48]. There may be a dose–response relationship, with higher-dose interventions (≥100 mg isoflavones/day) having slightly higher cognition performance than lower-dose ones [48]. In our study, the fermented soy intervention contained 36.3 mg isoflavones/day, which would be classified as low dose by this meta-analysis. However, most of those RCTs studied isoflavone supplements. It is estimated that not more than 10% of people in Asia consume 100 mg of isoflavones per day [41]. Older Japanese adults consume an estimated average of 25–50 mg isoflavones/day [41], thus our intervention may be considered moderate intake. Additionally, fermentation has been shown to increase human urinary excretion and recovery of aglycone isoflavones compared to unfermented soy, demonstrating improved bioavailability [49]. The enhanced bioavailability of fermented soy supports its potential for physiological benefit even at moderate intake levels.

Fermented soy may support cognition through multiple complementary mechanisms. Aglycone isoflavones, peptides, and amino acids derived from fermentation have demonstrated antioxidant and anti-inflammatory activity, helping to counter oxidative stress, an important contributor to neurodegenerative diseases of aging [15,19,50,51,52]. For example, fermented soy products such as tempeh have been shown in animal models to enhance memory by promoting antioxidant enzyme expression and reducing neuroinflammation. [20,21]. Additionally, animal and in vitro studies have demonstrated antihypertensive and ACE-inhibitory properties of soy peptides, which may improve vascular function and indirectly support brain health, given the link between hypertension and dementia risk [50,53].

Isoflavones also interact with estrogen receptor beta (ERβ), which is abundant in the hippocampus and cerebellum, key regions for memory and learning [54,55]. Activation of ERβ may contribute to the memory-specific effects observed in some soy studies. Finally, age-related changes such as increased oxidative stress, inflammation, and alterations in lipid metabolism and gut microbiota are known risk factors for cognitive decline. Fermented soy products like Q-Can Plus^®^ have demonstrated the ability to favorably modulate these pathways [29,30,35], offering a potential preventive benefit for ARCD if administered before irreversible damage occurs.

To the best of our knowledge, this is the first RCT to investigate the effects of a non-probiotic, fermented soy product in a group of cognitively healthy older adults on global and domain-specific cognition. Strengths of this study include the use of a triple-blind, placebo-controlled, randomized design and the use of a broad battery of standardized cognitive tests across different domains of cognition, successfully used by us in previous trials with older adults [42]. One of the limitations of the study was that there were only 47 completers, mostly women ≥ 70 years, limiting statistical power and external validity. We acknowledge that the improvements in global cognition and memory scores observed among women aged 70 years and older were modest, and, as they emerged from exploratory analyses, should be interpreted with caution. Future studies are needed to confirm these findings. We also had a higher drop-out rate in this study compared to our previous trial using this fermented soy powder [29]. Of those who dropped out, six participants were under 70 years old, and significantly more Hispanics dropped out compared to other racial groups, which may have introduced bias. The Hispanic population is typically underrepresented in clinical trials, thus particular attention to recruitment and retention should be a priority in follow-up studies [56]. Since this was a 12-week intervention, only short-term effects on cognition could be observed. Our study was also limited by only utilizing subjective assessment of intake and did not include a biological biomarker of soy intake. Future studies should consider longer term intervention in larger samples, with biomarkers such as BDNF and isoflavones to support and further confirm our observations, clarify potential causes of age- and gender-related differences in outcomes, as well as consider the effect on preclinical markers of Alzheimer’s disease.

## 5. Conclusions

We demonstrated that fermented soy improves memory in older adults, with stronger improvements in memory and global cognition observed in postmenopausal women 70 years and older. These findings are hypothesis-generating; thus, causality cannot be inferred, especially given the small sample and exploratory subgroup analysis.

Memory is crucial for daily decision making and functioning, and thus regular intake of fermented soy could be a cost-effective way to delay cognitive decline. With prior research supporting the cholesterol-lowering, antioxidant, anti-inflammatory, and gut health effects of Q-Can Plus^®^, there may be the potential to consider fermented soy as part of a diet that can preserve both heart health and cognition.

## Figures and Tables

**Table 1:** Baseline characteristics of fermented soy and placebo groups ^1^.

	Fermented Soy	Placebo	*p*-Value
	(*n* = 25)	(*n* = 22)	
Sex			.
Female, *n* (%)	18 (72%)	18 (82%)	0.505 ^2^
Age, y	74.1 ± 4.5	74.0 ± 5.7	0.911 ^3^
Female ≥ 70 y, *n* (%)	16 (64%)	13 (59%)	0.482 ^2^
Race/ethnicity, *n* (%)			0.962 ^4^
Caucasian/White	17 (68%)	15 (68%)	
Hispanic	4 (16%)	3 (14%)	
Other	4 (16%)	4 (18%)	
Education, number of y	15.8 ± 2.2	16.2 ± 1.80	0.429 ^3^
More than 12 y, *n* (%)	24 (96%)	23 (100%)	
Height, cm	166.0 ± 11.4	165.0 ± 8.5	0.753 ^3^
Weight, kg	81.5 ± 19.1	74.1 ± 13.9	0.145 ^3^
BMI	29.4 ± 5.5	27.2 ± 5.0	0.164 ^3^
Coffee consumption, *n* (%)			0.536 ^4^
None	13 (52%)	10 (46%)	
Low	12 (48%)	11 (50%)	
Moderate	0 (0%)	1 (5%)	
Alcohol consumption ^5^, *n* (%)			0.136 ^4^
None	14 (58%)	7 (33%)	
Consumers	10 (42%)	14 (67%)	
Never smoker ^5^, *n* (%)	17 (71%)	16 (76%)	0.746 ^2^
Physical activity ^5^, *n* (%)			0.935 ^4^
Sedentary	5 (21%)	2 (9%)	
Light	4 (17%)	9 (41%)	
Moderate	15 (62%)	11 (50%)	
Center for Epidemiological Studies Depression Scale: 0–60 scale	9.1 ± 7.1	10.5 ± 9.1	0.576 ^3^
At risk of depression: score ≥ 16, *n* (%)	6 (24%)	4 (18%)	0.451 ^2^
Stress, perceived, *n* (%)			0.770 ^4^
Low	13 (52%)	13 (59%)	
Moderate to high	12 (48%)	9 (41%)	

^1^ Values are *n* (%) or mean ± SD. ^2^ Fisher’s exact test. ^3^ Independent *t*-test. ^4^ Pearson chi-square test. ^5^ Fermented soy group, *n* = 24; Placebo group, *n* = 21.

**Table 2:** Cognitive composite scores of fermented soy and placebo groups ^1,2^.

Cognitive Composites	Fermented Soy	Placebo	*p* Value
	(*n* = 25)	(*n* = 22)	
Global cognition			
Baseline	57.83 (54.51, 61.15)	57.71 (54.17, 61.25)	0.961
12 weeks	59.66 (55.95, 63.37)	58.85 (54.90, 62.81)	0.765
Unadjusted change	1.84 (0.41, 3.26)	1.15 (−0.37, 2.66)	0.507
Adjusted change ^3^	1.06 (−0.48, 2.60)	0.86 (−1.05, 2.78)	0.200
Memory			
Baseline	57.91 (52.38 63.43)	52.34 (46.45, 58.22)	0.172
12 weeks	61.62 (55.75, 67.49)	55.23 (48.97, 61.48)	0.140
Unadjusted change	3.71 (−0.01, 7.44)	2.89 (−1.08, 6.86)	0.763
Adjusted change ^3^	1.81 (−2.10, 5.72)	1.16 (−3.64, 5.97)	0.041
Verbal fluency			
Baseline	42.43 (38.69, 46.17)	42.52 (38.54, 46.51)	0.973
12 weeks	42.31 (38.12, 46.50)	44.26 (39.80, 48.73)	0.524
Unadjusted change	−0.12 (−2.23, 1.99)	1.74 (−0.51, 3.99)	0.231
Adjusted change ^3^	−0.15 (−2.57, 2.27)	2.44 (−0.56, 5.45)	0.720
Processing speed			
Baseline	63.94 (60.10, 67.79)	67.21 (63.12, 71.31)	0.247
12 weeks	65.96 (62.02, 69.90)	67.77 (63.58, 71.97)	0.528
Unadjusted change	2.02 (0.11, 3.93)	0.56 (−1.48, 2.59)	0.298
Adjusted change ^3^	1.51 (0.57, 3.77)	0.37 (−2.29, 3.03)	0.478
Executive function			
Baseline	67.03 (62.84, 71.22)	69.76 (64.29, 73.22)	0.573
12 weeks	68.76 (64.18, 73.34)	68.15 (63.26, 73.03)	0.855
Unadjusted change	1.73 (−0.27, 3.73)	−0.61 (−2.74, 1.52)	0.114
Adjusted change ^3^	1.28 (−1.02, 3.57)	−0.85 (−3.70, 2.00)	0.506

^1^ Percent of maximum possible. ^2^ Mean (95% Confidence Interval). ^3^ Corrected for sex and baseline scores using ANCOVA.

**Table 3:** Raw test score changes for fermented soy and placebo groups ^1,2^.

	Fermented Soy Group (*n* = 25)	Placebo Group (*n* = 22)	*p*-Value
Memory			
Rey Auditory Verbal Learning Test, immediate recall	−0.08 (9.25)	−0.36 (7.22)	0.908
Rey Auditory Verbal Learning Test, delayed recall	−0.24 (2.19)	0.50 (2.82)	0.317
Brief Visuospatial Memory Test-Revised, immediate recall	2.84 (4.51)	1.50 (4.03)	0.291
Brief Visuospatial Memory Test-Revised, delayed recall	1.04 (2.30)	0.55 (1.50)	0.395
Verbal Fluency			
FAS Test	1.16 (6.49)	0.64 (6.33)	0.781
Animals Naming Test	−0.56 (2.93)	1.14 (2.93)	0.054
Processing Speed			
Symbol Digit Modalities Test	2.48 (5.68)	2.86 (4.98)	0.808
Trail Making Test A ^3^	−2.96 (7.57)	−1.72 (9.09)	0.611
Stroop Word	0.40 (8.58)	−2.18 (6.99)	0.268
Stroop Color	1.20 (5.61)	−0.82 (6.08)	0.243
Executive Function			
Trail Making Test B ^3^	−7.01 (21.99)	11.31 (47.13)	0.088
Stroop Color and Word	1.32 (4.96)	−0.91 (4.83)	0.127
Digit Span Test	−0.32 (3.01)	−0.09 (2.93)	0.793
Automated Cognitive Test	1.63 (4.65)	2.27 (5.57)	0.671

^1^ Mean (SD). ^2^ Independent samples *t*-test, *p* < 0.05. ^3^ Score is seconds taken to complete test.

## Data Availability

The data presented in this study are available on request from the corresponding author. The data are not publicly available due to ethical reasons.

## Supplementary Materials

The following supporting information can be downloaded at: https://www.mdpi.com/article/10.3390/nu17182936/s1, Table S1: Baseline characteristics of completers and dropouts; Table S2: Baseline raw test scores of fermented soy and placebo groups; Table S3: Cognitive composite scores of fermented soy and placebo groups for women 70 y and older.

## Abbreviations

The following abbreviations are used in this manuscript:

ARCD	Age-related cognitive decline
MCI	Mild cognitive impairment
BDNF	Brain-derived neurotrophic factor
RCT	Randomized, controlled trial
TMT	Trail Making Test
ANCOVA	Analysis of covariance
ERβ	Estrogen receptor beta
