# Supplementary material for: Effects of Fermented Soy on Cognition in Older Adults: Outcomes of a Randomized, Controlled Trial
Source: Nutrients. 2025 Sep 12;17(18):2936. doi: 10.3390/nu17182936 (PMC12472887; doi:10.3390/nu17182936)
Supplement: Supplementary file 1 [file nutrients-17-02936-s001.zip › nutrients-3790770-supplementary.pdf]

nutrients MDPI

Supplementary Material

Table S1. Baseline characteristics of completers and dropouts[1,2].

	Completers (n = 47)	Dropouts (n = 13)	*p*-Value
	(*n* = 47)	(*n* = 13)	
Sex			.
Female, *n* (%)	36 (77%)	9 (69%)	0415[2]
Age, y	74.0 ± 5.0	71.7 ± 5.5	0.148[3]
Female ≥ 70 y, *n* (%)	29 (62%)	5 (39%)	0.206[2]
Race/ethnicity, *n* (%)			0.013[4]
Caucasian/White	32 (68%)	5 (38%)	
Hispanic	7 (15%)	7 (54%)	
Other	8 (17%)	1 (8%)	
Education, y	15.8 ± 2.2	16.2 ± 1.80	0.429[3]
> 12 y, *n* (%)	24 (96%)	23 (100%)	
BMI	28.4 ± 5.51	28.5 ± 5.2	0.957[3]
Never smoker[5], *n* (%)	32 (73%)	9 (75%)	0.595[2]
Physical activity, *n* (%)[5]			0.219[4]
Sedentary	8 (18%)	5 (42%)	
Light	13 (30%)	2 (17%)	
Moderate	23 (52%)	5 (42%)	
Center for Epidemiological Studies Depression Scale: 0-60 scale	11.8 ± 5.7	9.7 ± 8.0	0.399[3]
At risk of depression: score ≥ 16, *n* (%)	10 (21%)	3 (23%)	0.578[2]
Stress, perceived, *n* (%)			0.925[4]
Low	26 (55%)	7 (54%)	
Moderate to high	21 (45%)	6 (46%)	
Global cognition composite, *n* (%)[6]	57.8 (8.2)	56.5 (9.3)	0.637

[1]Values are *n* (%) or mean ± SD [2]Fisher's exact test. [3]Independent *t*-test. [4]Pearson chi-square test. [5]Completers, n = 44; Dropouts, n = 12. [6]Percent of maximum possible.

Table S2. Baseline raw test scores of fermented soy and placebo groups[1,2].

	Fermented soy group	Placebo group	*p*-Value
	(*n* = 25)	(*n* = 22)	
**Memory**			
Rey Auditory Verbal Learning Test, immediate recall	41.80 ± 11.23	40.59 ± 9.39	0.693
Rey Auditory Verbal Learning Test, delayed recall	8.20 ± 3.01	6.64 ± 3.09	0.086

Brief Visuospatial Memory Test-Revised, immediate recall	19.76 ± 5.36	17.73 ± 6.06	0.229
Brief Visuospatial Memory Test-Revised, delayed recall	7.96 ± 2.30	7.41 ± 2.22	0.409
**Verbal Fluency**			
FAS Test	37.76 ± 11.07	40.95 ± 12.51	0.358
Animals Naming Test	18.84 ± 4.86	17.64 ± 3.82	0.355
**Processing Speed**			
Symbol Digit Modalities Test	39.76 ± 7.95	39.82 ± 9.23	0.982
Trail Making Test A[3]	31.12 ± 15.51	29.34 ± 9.66	0.643
Stroop Word	86.16 ± 13.72	93.18 ± 12.85	0.078
Stroop Color	60.24 ± 9.80	64.41 ± 11.59	0.188
**Executive Function**			
Trail Making Test B[3]	94.97 ± 53.70	83.59 ± 33.44	0.396
Stroop Color and Word	32.80 ± 6.18	34.14 ± 9.06	0.553
Digit Span Test	26.84 ± 4.24	27.14 ± 4.66	0.821
Automated Cognitive Test	42.40 ± 7.15	43.09 ± 8.22	0.761

[1]Mean ± SD.

[2]Independent samples T-test, $p < 0.05$.

[3]Score in seconds taken to complete test

Table S3. Cognitive composite scores of fermented soy and placebo groups for women 70 years and older[1,2].

**Cognitive composites**	**Fermented soy (n = 16)**	**Placebo (n = 13)**	***P* value**
**Global cognition**			
Baseline	56.90 (52.93, 60.87)	57.54 (53.13,61.94)	0.827
12 weeks	59.77 (55.60, 63.93)	57.60 (52.98, 62.22)	0.481
Unadjusted change	2.87 (1.55, 4.18)	0.60 (-1.63, 1.43)	0.007
Adjusted change[3]	2.86 (1.52, 4.21)	0.62 (-1.40, 1.52)	0.028
**Memory**			
Baseline	53.51 (47.19, 59.82)	52.82 (43.74, 61.89)	0.891
12 weeks	61.96 (53.88, 70.05)	54.88 (46.86, 62.90)	0.198
Unadjusted change	8.46 (5.09, 11.82)	2.06 (-1.67, 5.79)	0.015
Adjusted change[3]	8.47 (5.05, 11.89)	2.05 (-1.75, 5.84)	0.049
**Verbal fluency**			
Baseline	41.09 (36.35, 45.84)	41.37 (36.10, 46.63)	0.938
12 weeks	40.98 (35.93, 46.04)	40.19 (34.59, 45.80)	0.831
Unadjusted change	-0.11 (-2.79, 2.58)	-1.17 (-4.15, 1.80)	0.591
Adjusted change[3]	-0.12 (-2.82, 2.58)	-1.16 (-4.16, 1.84)	0.604
**Processing speed**			
Baseline	65.19 (60.98, 69.39)	68.21 (63.11, 73.32)	0.328

12 weeks	66.74 (63.13, 70.35)	68.86 (63.21, 74.51)	0.485
Unadjusted change	1.55 (-0.54, 3.64)	0.65 (-1.68, 2.97)	0.558
Adjusted change[3]	1.36 (-0.71, 3.43)	0.88 (-1.42, 3.18)	0.294
**Executive Function**			
Baseline	67.82 (62.66, 72.98)	67.76 (62.04, 73.48)	0.987
12 weeks	69.39 (63.65, 75.12)	66.46 (60.10, 72.83)	0.490
Unadjusted change	1.57 (-1.12, 4.25)	-1.30 (-4.27, 1.68)	0.154
Adjusted change[3]	1.57 (-1.17, 4.30)	-1.30 (-4.33, 1.74)	0.365

[1]POMS = Percent of maximum scaling

[2]Mean (95% Confidence Interval).

[3]Adjusted change corrected for baseline scores using ANCOVA.
